# Carrageenan–Acyclovir Complex and Its Liposomal Form

**DOI:** 10.3390/ijms27083367

**Published:** 2026-04-09

**Authors:** Irina M. Yermak, Valery P. Glazunov, Natalya V. Krylova, Vladimir I. Gorbach, Anna O. Kravchenko, Alexandra V. Volod’ko, Viktoriya N. Davydova, Dmitry K. Chistyulin, Mikhail Y. Shchelkanov

**Affiliations:** 1 G.B. Elyakov Pacific Institute of Bioorganic Chemistry, Far-Eastern Branch of the Russian Academy of Sciences (FEB RAS), 100 Let Vladivostoku Prosp. 159, 690022 Vladivostok, Russia; glazunov@piboc.dvo.ru (V.P.G.); vigorbach@bk.ru (V.I.G.); kravchenko_25.89@mail.ru (A.O.K.); morskaia@list.ru (A.V.V.); vikdavidova@yandex.ru (V.N.D.); cdk27@mail.ru (D.K.C.); 2 G.P. Somov Institute of Epidemiology and Microbiology, Rospotrebnadzor, Selskya St. 1, 690087 Vladivostok, Russia; krylovanatalya@gmail.com (N.V.K.); adorob@mail.ru (M.Y.S.)

**Keywords:** carrageenan, acyclovir, liposomes, quantum-chemical calculations, anticherpetic activity

## Abstract

Sulfated polysaccharides, carrageenans (CRGs) derived from *Chondrus armatus*, were used as mucoadhesive matrices for incorporating the antiviral drug acyclovir (ACV). Through IR spectroscopy and quantum-chemical calculations, it was demonstrated that CRGs interact with ACV, forming complexes via intermolecular hydrogen bonding and coordination interactions, with an enthalpy of approximately 15–20 kcal/mol. The monomodal ζ-potential distribution observed in the CRG/ACV mixture confirmed the successful formation of this complex. The antiviral efficacy of the CRG/ACV complex was evaluated during the early stages of herpes simple virus (HSV-1) infection, focusing on its ability to inhibit the cytopathic effects of the virus on host cells. Notably, CRGs enhanced antiviral activity by allowing a reduction in the ACV dosage. Unlike ACV alone, the CRG complex exhibited a prophylactic effect, with therapeutic efficacy comparable to that of ACV. When incorporated into liposomes, the CRG/ACV complex displayed excellent mucoadhesive properties. This liposomal formulation demonstrated notable antiviral activity against infected cells with selective index (SI) 344 and a heightened prophylactic effect (SI 128) compared to the complex alone. Overall, these new antiviral compounds show promise in selectively inhibiting viral adsorption and replication processes without adversely affecting the host organism.

## 1. Introduction

Sulfated polysaccharides play a crucial role in the medical and pharmaceutical sectors and are recognized for their diverse biological activities, biocompatibility, biodegradability, and mechanical strength. Among algal polysaccharides, carrageenans (CRGs) derived from red seaweeds have been the focus of extensive research regarding their safety profiles in terms of toxicity, pyrogenicity, and allergenicity. These compounds exhibit a broad range of biological functions, including immunomodulatory, anticancer, anticoagulant, and antiviral effects, which are closely linked to their structural characteristics [1,2]. The primary backbone structure of CRGs is characterized by alternating 3-linked β-D-galactopyranose and 4-linked α-D-galactopyranose units. Various CRG types can be classified based on the configuration of the repeating disaccharide units, the pattern of sulfation, and the inclusion of 3,6-anhydrogalactose as a 4-linked residue. The most commonly utilized variants of CRGs in industrial contexts include kappa (κ-), iota (ι-), and lambda (λ-) CRGs, which differ in the number of sulfate groups per disaccharide unit; kappa has one sulfate group, iota has two, and lambda features three [3]. Extensive in vivo investigations of CRGs by multiple research teams have substantiated their safety for use in food and medical applications. Currently, CRG has been incorporated into the United States Pharmacopeia 35 National Formulary 30S1 (USP35-NF30S1), the British Pharmacopoeia 2012 (BP 2012), and the European Pharmacopoeia 7.0 (EP 7.0), indicating its promising potential as a pharmaceutical excipient. The wide array of potential therapeutic applications for various CRG types has sparked considerable interest in these polysaccharides [4,5,6,7]. CRGs show considerable promise in combating viral infections, particularly through their ability to impede viral entry and replication [7,8]. Research indicates that CRGs are effective against a variety of enveloped RNA and DNA viruses, including herpesviruses (HSVs), which are among the most prevalent viral pathogens affecting humans [9]. These sulfated polysaccharides can be integrated into pharmaceutical formulations aimed at preventing or treating viral illnesses without associated side effects [10]. Notably, a CRG-based gel has been shown to offer protection against HSV-2 by binding to the viral receptors, thereby obstructing the viral ability to attach to host cells [11].

The current benchmark for treating herpes virus infections continues to be acyclovir (ACV), prized for its high selectivity. Nevertheless, extensive evidence has emerged highlighting the resistance of HSV-1 to numerous nucleoside analogue-based therapies, including both ACV and its derivatives [12]. Additionally, ACV presents several drawbacks: it has low bioavailability, limited solubility, requires large doses, and necessitates frequent administration, all of which contribute to its toxicity [13]. ACV is commonly used to manage recurrent herpes simplex on the lips and face, genital herpes, herpes zoster, and chickenpox, with administration via oral, parenteral, and topical routes. However, the parenteral route is restricted by side effects, while topical applications have proven ineffective due to the drug’s low skin permeability [14]. The oral bioavailability of ACV is limited (15–30%) because of inconsistent and incomplete absorption, primarily attributed to its inability to effectively permeate the gastrointestinal membrane [15]. To mitigate the drug’s toxicity and enhance targeted delivery, stable nanoliposomal and nano-niosomal formulations of ACV have been developed, designed to maintain a prolonged presence in systemic circulation [16]. Arijit Gandhi and colleagues [17] pursued the creation of polymeric nanoparticles for ACV to optimize delivery and address its inherent limitations. Their research demonstrated that Eudragit nanoparticles effectively sustained drug release over extended periods.

Mucoadhesives play an important role in increasing the bioavailability of the active substance by increasing its residence time at the delivery site. The combination of the carbomer 934P and Azadirachta fruit mucus were developed for the controlled release of ACV in order to obtain mucoadhesive microspheres [18]. The authors convincingly demonstrated that the mucoadhesive properties of Azadirachta fruit mucus enhanced ACV encapsulation and ensured its effective delivery to the stomach.

Among mucoadhesive substances, hydrophilic biopolymers such as carrageenans have attracted particular attention as transport systems for drug delivery [6,7,19,20]. The exceptional water-absorption capacity of CRG significantly increases the solubility of drugs, thereby improving the oral bioavailability of poorly water-soluble compounds. For instance, the solubility of tolterodine ι-tartrate was effectively increased through its complexation with λ-CRG [21]. Furthermore, by combining various polymers, including CRG, researchers have developed efficient mucoadhesive systems that can modulate the release of ACV. One notable example is the combination of CRG with poloxamer 407 to create a thermosensitive gel suitable for vaginal administration of ACV, which has shown promising results [22]. To achieve controlled ACV release, solid vaginal formulations using marine polysaccharides have also been developed [23]. One study demonstrated that combining chitosan or κ-CRG with the semi-synthetic polymer hydroxypropyl methylcellulose in solid compact forms can facilitate sustained release of ACV.

In these experimental works, carrageenan was primarily considered a carrier for the prolonged action of ACV. Despite the extensive use of CRG in various formulations for ACV delivery, the interactions between the drug and polymer carrier remain largely unexplored. The presence of diverse functional groups in CRG may facilitate interaction with the drug, and understanding the nature of these interactions is crucial not only for enhancing adsorption capacity and achieving sustained release, but also for preventing conjugation or covalent modifications that could alter drug properties. Moreover, the impact of the polymer carrier on the antiviral activity of ACV remains insufficiently studied. It is essential to assess the overall antiviral efficacy of polymer systems containing ACV, especially considering that while ACV is recognized for inhibiting herpes simplex virus-1 by interfering with DNA synthesis and blocking viral replication, it does not prevent viral attachment.

In our previous research, we investigated the antiviral properties of CRGs extracted from red algae along the Pacific coast, focusing on their efficacy against HSV-1. Our findings highlighted a correlation between the structural characteristics of these polysaccharides and their antiviral activity [24]. Specifically, CRGs containing DA units (3,6-anhydrogalactose) demonstrated a significant ability to inhibit viral binding to host cells and exhibited strong anti-HSV-1 activity during the initial stages of viral attachment. Pre-treatment of Vero cells with these polysaccharides prior to viral infection revealed a noteworthy reduction in HSV-1 plaque formation (SI 111), while ACV showed no preventive effect. Combining CRG and ACV may create a synergistic effect, enhancing protection against the virus at multiple stages of the infection process. We have previously established that CRGs possess excellent mucoadhesive properties [25], which could be beneficial for localized ACV delivery, as well as for oral administration. These routes of administration that are mainly used to treat infections caused by the herpes simplex virus (HSV-1/2). In recent years, liposomal drug delivery systems have gained popularity for their ability to enhance the efficacy and specificity of pharmaceuticals while minimizing toxicity. Incorporating drugs into liposomes facilitates localized and sustained delivery to mucosal surfaces [26].

The purpose of this work was to explore the possibility of obtaining an active CRG/ACV complex capable of enhancing the effect of the antiviral drug, including by reducing its dose and incorporating it into liposomes to provide an effective drug delivery system. Additionally, one of the main aspects of this work is devoted to studying the interaction between CRG and ACV, which may be an important step selecting a polymer carrier for ACV.

## 2. Results

### 2.1. Polysaccharide Characteristics

CRGs were extracted from the red seaweed *Chondrus armatus*, purified to eliminate low-molecular-weight impurities, and precipitated using alcohol, yielding a total polysaccharide fraction of 50%. This highly purified polysaccharide was named Σ-CRG. The Σ-CRG was then fractionated with 4% potassium chloride into gelling (KCl-insoluble) and non-gelling (KCl-soluble) fractions. The structure of the gelling polysaccharide was analyzed using NMR and FTIR spectroscopy, with results compared to previous studies [27]. The IR absorption bands and NMR chemical shifts were identified by comparison with established carrageenan structures [3]. In the IR spectra, a strong absorption band at 1236 cm^−1^ indicated a presence of high sulfate groups, consistent with chemical analyses (Figure S1). Characteristic absorption bands at 930 and 847 cm^−1^ suggested the presence of 3,6-anhydrogalactose and a secondary sulfate group, assigning the polysaccharide as κ-CRG.

^13^C NMR spectroscopy further confirmed the FTIR findings, identifying specific signals corresponding to 3-linked β-D-galactose (103.1 ppm) and 4-linked 3,6-anhydro-α-D-galactose (95.9 ppm) (Supplementary Table S1). In the ^1^H NMR spectrum, signals were observed in the region of anomeric protons at 4.6 and 5.1 ppm, corresponding to the 3-linked residue of β-D-galactose and C-1of 4-linked 3,6-anhydro-α-D-galactose. Thus, the gelling polysaccharide was identified as κ-CRG, aligning with prior research. According to HPLC the average molecular weight of κ-CRG was 560 kDa. The non-gelling KCl-soluble fraction corresponded to λ-type CRG [28]. The IR-spectrum of Σ-CRG (Supplementary Figure S2) contained both absorption bands characteristic of κ-CRG and λ-CRG [3,27]. However, the ^13^C NMR spectra of Σ-CRG could not be resolved due to high viscosity and a disordered structure. Partial reductive hydrolysis revealed that the Σ-CRG consisted solely of carrabiose.

According to HPLC, Σ-CRG was quite heterogeneous in molecular weight, which averaged 180 kDa. So, the unfractionated Σ-CRG containing both κ- and λ-CRGs was used. The structures of repeating disaccharide units and molecular weights are listed in Supplementary Table S2.

### 2.2. The Interaction of CRG with ACV

#### 2.2.1. FTIR Analysis

**Figure 1:**
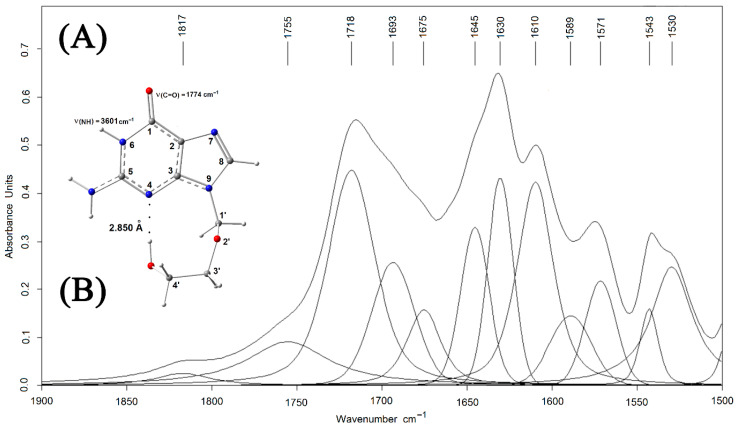
(**A**)—Fragment of the IR spectrum of crystalline acyclovir with the results of its decomposition into individual components (in KBr); (**B**)—molecular structure of ACV.

To study the possibility of binding between CRG and ACV, FTIR spectra of ACV, CRG, and CRG/ACV were obtained. Infrared spectroscopy is a rapid and non-destructive technique that has been widely used to characterize different polysaccharides. Decomposition of the IR spectrum of crystalline ACV into individual components in the region of 1900–1500 cm^−1^ (Figure 1) shows the presence of three individual absorption bands at 1718, 1693, and 1675 cm^−1^.

According to the literature data, the highest-frequency and most intense band at 1718 cm^−1^ belongs to the stretching vibration of the carbonyl group of ACV [29], while the other two bands (at 1693 and 1675 cm^−1^) may correspond to the stretching vibrations of the carbonyl group of one ACV molecule bound by intermolecular hydrogen bonds (IMHBs) with the N(6)−H or NH_2_ group of another ACV molecule [30].

**Figure 2:**
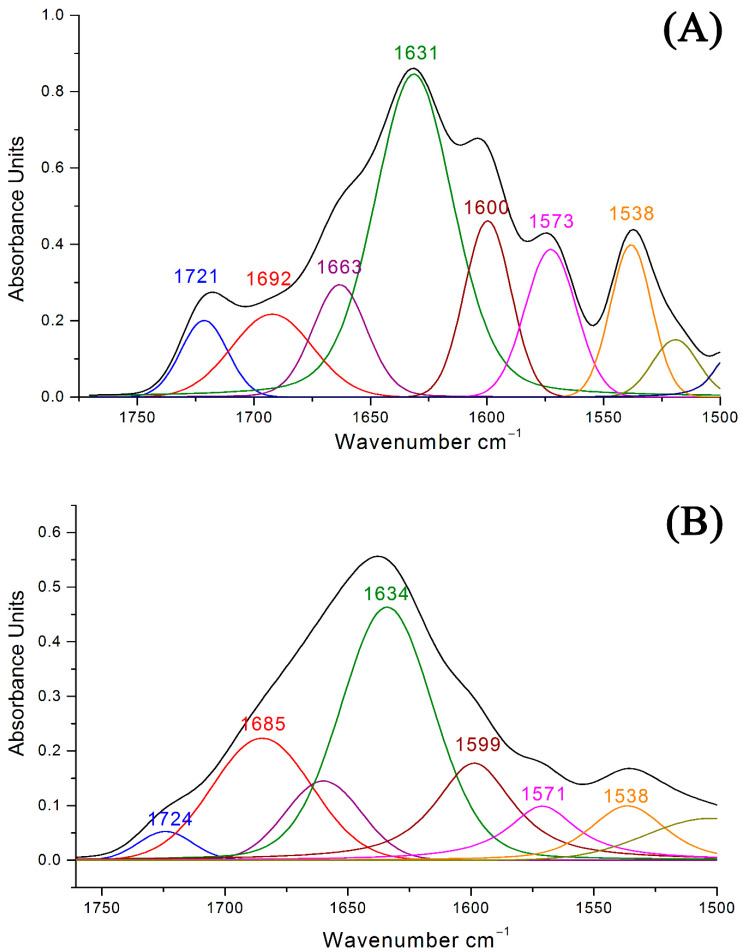
Decomposition of a fragment of the IR spectrum of the κ-CRG + ACV film into individual bands at different molar ratios of the components: (**A**)—7.5:1; (**B**)—30:1.

To demonstrate the interaction of κ-CRG with ACV, films of their mixtures with different ratios of the initial components were prepared and studied by IR spectroscopy. As shown in Figure 2, in the IR spectra of CRG/ACV films, the intensity at the maximum of the absorption band ν(C=O) at 1721 (1724 cm^−1^) decreased with an increasing κ-CRG/ACV concentration ratio relative to the band at 1634 (1631 cm^−1^) (Figure 2A,B), selected as an internal “standard”. This decrease may be attributed to the formation of intermolecular hydrogen bonds between ACV and κ-CRG, as indicated by the shift of the maximum of this band toward shorter wavelengths by 29–39 cm^−1^. The molar concentration of κ-CRG was calculated based on the molecular weight of the disaccharide unit containing the Na^+^ cation.

#### 2.2.2. Quantum-Chemical Calculations to Evaluate the Binding of CRG with ACV

**Figure 3:**
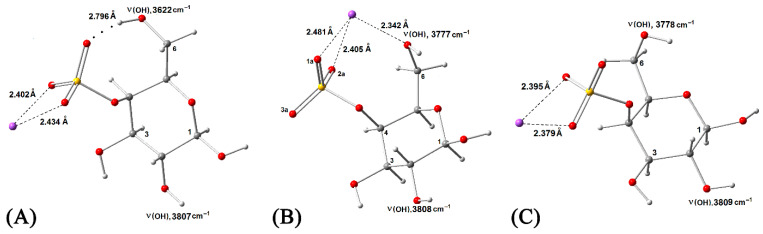
Different conformers (**A**–**C**) of monosaccharide residue of κ-CRG.

In this work, quantum-chemical calculations were used to confirm complex formation and determine the enthalpy of interaction between CRG and ACV. For this purpose, the monosaccharide residue of the disaccharide unit of κ-CRG—(1 → 3) 4-O-sulfate-*β*-D-galactopyronosyl was used. This residue contains two OH groups at C(2) and C(6), and one sulfate group at C(4) in neutral form SO_3_^−^Na^+^, which can participate in the interaction of CRG with ACV. Additionally, the sodium cation in the sulfate group of the κ-CRG monosaccharide residue can form coordination bonds (CBs) with the oxygen atoms of the carbonyl group or the nitrogen atoms of ACV. It is known that the first coordination shell of Na^+^ can include up to six water molecules. In such a cluster, Na^+^ forms six CBs with equidistant oxygen atoms of water molecules, R(Na^+^ → O) = 2.452 Å [31]. The formation of such CBs must be taken into account in the conformational analysis of κ-CRG/ACV. In addition to the carbonyl group, the ACV molecule contains an N(6)—H group, which can participate in the formation of intermolecular hydrogen bonds (IMHBs). The monosaccharide residue of κ-CRG can be represented by different conformers. The most stable conformer, which has the maximum total Gibbs energy in absolute value (main conformer), contains two CBs and one intramolecular hydrogen bond between the O(6)—H group and the S=O bond of the sulfate group (Figure 3A).

**Figure 4:**
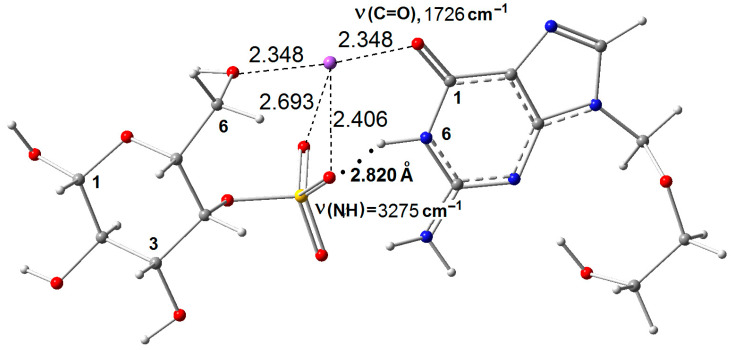
Theoretical model of the “main” ACV/κ-CRG complex with an intermolecular hydrogen bond N(6)—H…O=S and with four coordination bonds Na^+^ → O.

A comparison of the enthalpies of conformers (Figure 3A,C) made it possible to estimate the enthalpy of the IMHBs (−Δ*H*(IMHB) = 5.30 kcal/mol), while a comparison of Figure 3B,C allowed estimation of the enthalpy of the CBs (−Δ*H*(CB) = 5.10 kcal/mol). The second most stable conformer has three CBs (Figure 3B) but does not contain an intramolecular hydrogen bond. The conformational analysis carried out in this work showed that among the possible monomolecular complexes of κ-CRG with ACV (with IMHBs and CBs), the main complex with the maximum absolute value of total Gibbs energy was the complex shown in Figure 4. The enthalpy of this complex −Δ*H* = 13.6 kcal/mol was evaluated as the difference between the enthalpy complex κ-CRG/ACV and the sum of enthalpy reactants κ-CRG and ACV. In this complex, ACV forms IMHBs between the N(6)–H group of ACV and one S=O bond of CRG, and Na^+^ forms four CBs: one with the carbonyl group of ACV, two with two S=O bonds of the sulfate group, and one with the O(6)–H group.

In the theoretical spectra, the shift to the lower frequency of the stretching vibration of the carbonyl group of ACV upon complex formation with IMHBs is 33–48 cm^−1^, which is in good agreement with the data obtained after decomposing the experimental IR spectra of films of the κ-CRG/ACV complexes into individual components (Figure 1A). An increase in the ratio of the absorption band areas of carbonyl groups confirms the assignment of the absorption band at 1685–1692 cm^−1^ to ν(C=O) of the carbonyl group of bound IMHBs. The enthalpy of the complex of the monosaccharide residue of λ-CRG containing two sulfate groups with two ACV molecules was 19 kcal/mol because two very strong IMHBs are realized in this complex (Supplementary Figure S3).

### 2.3. Dynamic Light Scattering (DLS) and Electrophoretic Properties of the CRG/ACV Complex

In this work, two CRG samples were used: κ-CRG and Σ-CRG. The formation of complexes of κ-CRG with ACV was monitored by dynamic light scattering (DLS) and electrokinetic measurements. The κ-CRG solution was titrated with ACV, and solutions of CRG/ACV mixtures at different weight ratios of the initial components (from 6:1 to 100:1 κ-CRG/ACV (*w*/*w*)) were obtained. According to DLS data, CRGs form particles in solutions with a high degree of polydispersity, which is characteristic of linear polysaccharides. Taking into account cumulative analysis data, the addition of ACV to the CRG solution resulted in an increase in particle size (Z-average value) from 614 nm to 1076 and 1152 nm for 10:1 and 100:1 CRG/ACV mixtures, respectively. A monomodal zeta-potential distribution of particles was observed in solutions of κ-CRG with ACV at ratios of the initial components of 10:1 and 100:1 (CRG:ACV *w*/*w*), which may indicate the formation of macroparticles of CRG/ACV complexes (Supplementary Figure S4). According to electrokinetic measurements, κ-CRG had a negative charge, with a surface potential of −85.3 ± 0.8 mV, while ACV exhibited a charge of −6.9 ± 2.9 mV. The addition of ACV to the CRG solution led to the formation of complexes whose charge was not equal to the sum of the charges of the initial components. The absolute value of the negative charge (74.1 ± 0.8) for the κ-CRG/ACV complex (10:1) was lower than that of κ-CRG but much greater than that of ACV (Table 1). Σ-CRG was also used to obtain a complex with ACV. As in the case of κ-CRG, a monodisperse charge distribution was observed for Σ-CRG with ACV in solution at ratios of the initial components of 10:1 and 100:1. The particle size of Σ-CRG increased significantly upon addition of ACV. The results of ζ-potential measurements of CRGs and their complexes with ACV are presented in Table 1.

### 2.4. Mucoadhesive Properties of the CRG/ACV Complex

**Figure 5:**
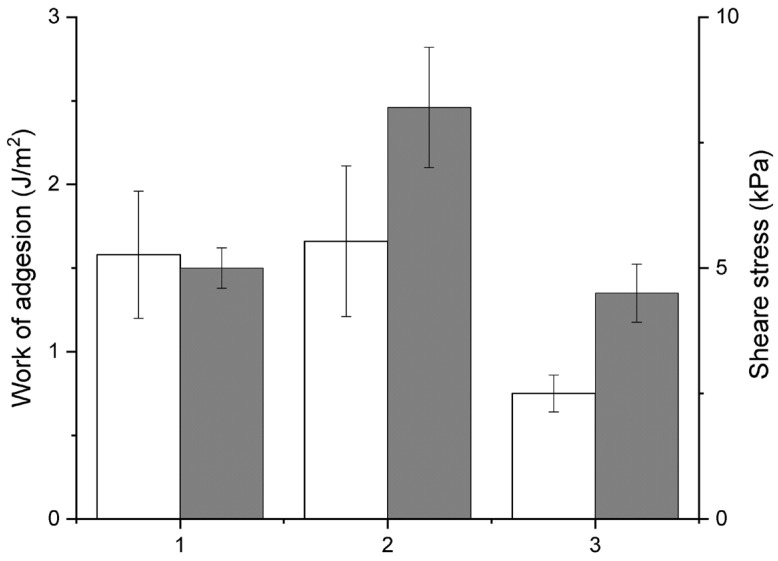
Work of adhesion (white column) and shear stress (grey): 1—CRG; 2—CRG/ACV complex; 3—control.

Mucoadhesion is frequently examined through in vitro experiments that explore potential interactions between samples and mucin at the molecular or colloidal scale before moving to ex vivo testing. The interaction between polymers and mucin may lead to the formation of aggregates with varying sizes and charges, which can be analyzed using different methods [32]. Previously, we demonstrated the mucoadhesive properties of CRGs using porcine mucin, which closely resembles human mucin [25]. By investigating their interaction with mucin, we can assess the mucoadhesive properties of the drug delivery system. The ability of the CRG/ACV complex to interact with mucin and display mucoadhesive characteristics was evaluated by measuring surface potential. Mucin bears a negative charge of −26.1 ± 0.8 mV, which is considerably lower in absolute value than that of CRG (−68.7 ± 0.7 mV). Introducing a mucin solution to the CRG/ACV complex reduced the CRG surface potential to −41.6 ± 0.7 mV. A single charge distribution was noted for the CRG/ACV and mucin mixtures, indicating their interaction (Table 1). Mucoadhesion is defined as the ability of materials to adhere to the soft mucosal surface that lines the gastrointestinal, tracheobronchial, reproductive, and human nasal system [20]. To determine the mucoadhesive properties of the CRG/ACV complex, a texture analyzer (Testing Machine EZ-LX, Shimadzu, Kyoto, Japan) was also used in accordance with the procedure described by Khutoryanskiy [33]. It is known that both the work of adhesion and peak peel force can be used to evaluate the mucoadhesiveness of potential adhesive polymer systems [34]. The mucoadhesive properties of the CRG/ACV complex were also assessed mechanically using a fresh-frozen porcine nasal mucosal tissue model. The adhesion was evaluated by the force of probe separation from the tissue after 60 s of pressing with a load of 0.01 N. As seen in Figure 5, the CRG/ACV complex exhibits similar or slightly greater mucoadhesive properties compared to the drug-free system.

### 2.5. Characterization of CRG/ACV-Containing Liposomes

Liposomes are a convenient form of drug delivery [35]. To obtain control (empty) liposomes, the standard thin-film hydration method followed by ultrasonic treatment was used. The Σ-CRG/ACV complex (10:1 *w*/*w*) was incorporated into liposomes as described in the Methods section, and the properties of the liposomes were examined by DLS and ζ-potential measurements. The particle sizes and ζ-potentials of the liposomes were measured immediately after sonication. Incorporation of CRG/ACV into the liposomes resulted in a slight increase in diameter compared to empty liposomes. For liposomes containing the CRG/ACV complex, a monomodal distribution of ζ-potential was observed. As shown in Table 1, liposomes containing CRG/ACV had a negative charge.

The obtained liposomes with CRG/ACV complex were lyophilized for storage and subsequent use. This procedure significantly extends the shelf life of the active substance, protects it from possible oxidation, and greatly facilitates transportation. Furthermore, lyophilization has several advantages over storing the active substance in a dissolved state, as it prevents degradation under the influence of factors in the dissolution medium and significantly reduces the risk of microbiological contamination. According to the data presented in Table 1, the size and *ζ*-potential of lyophilized liposomes containing CRG/ACV after storage for month and subsequent resuspension in pyrogen-free water were close to those of freshly prepared liposomes. This indicates that the liposomes retain their stability after lyophilization.

The mucoadhesive characteristics of liposomes were investigated by evaluating their interaction with mucin. The liposomes were incubated in a mucin solution for 2 h and analyzed using DLS. It was observed that the surface charge of mucin particles changed upon the addition of liposomes. The ζ-potential values of mucin particles increased from −26.1 ± 0.8 mV to −31.0 ± 0.6 mV in the presence of CRG/ACV-containing liposomes.

### 2.6. Antiherpetic Activity of CRG/ACV Complex and Its Liposome Form

For the combination of CRG with ACV, a complex based on Σ-CRG was selected, and a comparative in vitro assessment of its effectiveness relative to ACV was performed. The cytotoxicity of Σ-CRG, ACV, and the CRG/ACV complex toward Vero cells was assessed using the MTT assay. The studied compounds were non-toxic to the Vero cell culture: for Σ-CRG, CC_50_ values were >2000 μg/mL, while for ACV and the CRG/ACV complex, CC_50_ were >1000 μg/mL (Table 2). Determination of antiviral activity was carried out at concentrations below their CC_50_ values. The antiviral effect of the compounds at the early stages of HSV-1 infection was assessed by analyzing inhibition of the cytopathic effect (CPE) of the virus. To evaluate the inhibitory effect of Σ-CRG, ACV, and the CRG/ACV complex, both prophylactic (pre-treatment of cells with compounds) and therapeutic (treatment of infected cells) regimens were used (Table 2).

Exposure of Vero cells to the polysaccharide prior to HSV-1 infection (prophylactic effect) demonstrated that Σ-CRG exhibited considerable antiviral activity, with an inhibitory concentration of IC_50_ = 18 μg/mL. The selectivity index (SI = CC_50_/IC_50_), a measure of efficacy and safety, was notably high at 111. Conversely, Σ-CRG/ACV complexes (10:1 and 100:1 *w*/*w*) provided Vero cells with protection against herpesvirus infection, although the SI values were 3–4 times lower than those of Σ-CRG. Pre-treating cells with ACV did not influence viral replication.

Application of Σ-CRG after virus adsorption and cell penetration (1 h post-infection) proved significantly less effective than ACV, with the SI of Σ-CRG being 24 times lower than that of ACV. Treatment of infected cells using the Σ-CRG/ACV complex at a 100:1 ratio showed moderate activity (SI 63), while the 10:1 ratio exhibited activity comparable to ACV (SI 555 and 609, respectively).

Aqueous solutions of the Σ-CRG/ACV complex were incorporated into liposomes using the conventional thin-film method followed by sonication, and the antiviral efficacy of the liposomal form was evaluated. This complex was selected for liposome inclusion due to its antiviral properties, effectively protecting cells from cytopathic effects induced by HSV-1 and inhibiting early stages of viral replication. Prior to assessing antiviral activity, the cytotoxicity of both empty and complex-loaded liposomes toward Vero cells was evaluated. MTT assay results indicated low toxicity for both the liposomal form of the Σ-CRG/ACV complex and empty liposomes, with CC_50_ values exceeding 1000 μg/mL.

The antiviral activity of the liposomal form of the Σ-CRG/ACV complex against HSV-1, evaluated using the CPE inhibition assay, is summarized in Table 2. The findings reveal that the liposomal form demonstrated a markedly enhanced prophylactic effect compared to the complex itself, with a selectivity index (SI 189) more than five times higher. However, treatment of infected cells with Σ-CRG/ACV-loaded liposomes was less effective than the complex alone. Nevertheless, the elevated selectivity index (SI 322) indicates significant antiviral activity of liposomes containing the complex.

## 3. Discussion

Hydrophilic polysaccharides are traditionally used as mucoadhesive materials in many formulations for transmucosal drug delivery. The development of multifunctional drug delivery systems has become a relevant and attractive concept in pharmaceutical technology [36]. CRGs are ideal for non-invasive delivery of various drugs due to their biocompatibility, non-toxicity, and mucoadhesive properties [6,20]. Polymers placed on mucous membranes swell, resulting in the opening of a maximum number of adhesive sites, which provide interdiffusion and interpenetration of polymer chains and the mucin network [20,33]. In our work, we used CRG as a mucoadhesive matrix to incorporate the drug ACV, which is used in different forms for treating herpesvirus infection. Previously, we showed that CRGs isolated from the red algae *Chondrus armatus* had high antiviral activity against HSV [37]. The combined action of CRG and ACV could enhance the antiherpetic effect of the drug, reduce side effects, and decrease in the required dosage of ACV, and may be useful for both oral and topical administration. Although CRG is used in various drug delivery forms, including ACV, data on polysaccharide–drug interactions are lacking, as studies have mainly focused only on in vitro release. At the same time, the stability of such relatively simple drug delivery systems depends on the interaction of the drug with the polysaccharide matrix, since supramolecular bonds play a key role in various aspects of drug delivery, including biocompatibility, drug loading, and overcoming biological barriers [20,36]. Formulation of CRG with a drug provides controlled release and improves bioavailability [38]. For example, interaction between the nitrogen of trimetazidine hydrochloride and CRG contributed to the prolonged half-life of the drug–CRG conjugate [39], while interaction of carboxymethylated κ-CRG with insulin increased the amount of insulin in the polysaccharide matrix [40].

To assess the possible interaction of CRG with ACV, we used IR spectroscopy and quantum-chemical calculations. Decomposition of the IR spectra of CRG with ACV showed that interaction occurred due to the formation of intermolecular hydrogen bonds between CRG and ACV. Quantum-chemical calculations confirmed the formation of complexes involving intermolecular hydrogen bonds and coordination bonds, with enthalpy values reaching approximately 13–20 kcal/mol. The monomodal ζ-potential distribution observed for the CRG/ACV mixture also confirmed the formation of a complex between the polysaccharide and the drug. The decrease in CRG charge upon addition of ACV to its solution may result from their interaction. One of the important characteristics of drug delivery systems is their mucoadhesive properties [20,33,41]. The most important functional components of the mucous layer are mucin glycoproteins, which are responsible for the anti-adhesive properties of mucous membranes [33,34]. Studies of the interaction of natural polymers with mucins make it possible to predict the mucoadhesive properties of many dosage forms. The interaction of CRG/ACV with porcine stomach mucin was demonstrated by DLS. The increase in the ζ-potential of mucin observed after the addition of CRG/ACV complexes indicates the mucoadhesive properties of the latter. The data obtained by the mechanical method also showed that the inclusion of ACV polysaccharide matrix beads does not decrease the mucoadhesive properties of CRG (Figure 5). CRG systems containing ACV exhibit good mucoadhesive properties.

Viral infections such as herpes simplex virus remain a serious threat. The risk of developing resistance to antiviral drugs is increasing, and there is an urgent need for new antiviral agents. Previously, we studied the effect of CRG on different stages of viral infection and showed that κ-CRG containing DA units inhibits virus binding to cells and exhibits high anti-HSV-1 activity at the stage of viral attachment. Moreover, using molecular docking, we showed that CRGs are able to bind to the surface glycoprotein gD of the herpes virus type 1 (HSV-1), preventing the virus from interacting with cells [34].

At the same time, ACV exerts antiviral effects by preventing DNA synthesis and suppressing viral replication [42]. Considering the different effects of CRG and ACV at various stages of viral infection, we demonstrated the formation of CRG/ACV complexes to enhance antiviral activity and prophylactic action. The main disadvantages of ACV include low skin permeability, poor solubility, side effects, and the development of resistance of herpes viruses to ACV and its analogues. The interaction of CRG with ACV and the formation of a complex with a low drug content make it possible not only to improve the properties of ACV but also to maintain high antiviral activity. Treatment of Vero cells with both the polysaccharide and the CRG/ACV complex prior to HSV-1 infection demonstrated high antiviral activity, whereas ACV had no effect on viral replication. At the same time, the antiviral activity of the complex was comparable to that of ACV when treating infected cells. Thus, ACV in its new form suppresses viral attachment to cells and can be used not only for treatment but also for prevention of herpesvirus infections. Considering the high antiherpetic activity of the CRG/ACV complex due to the combined action of the components, we obtained a new type of this complex in the form of liposomes. The similarity of liposomes to cell membranes, along with their ability to incorporate the CRG/ACV complex without changing its chemical nature, makes liposomes a convenient drug delivery system. Selection index (SI) analysis in the Vero cell model indicates high potential for CRG/ACV complex and its liposomal form. According to the criteria accepted in pharmacology, compounds with SI > 10 are considered promising for further preclinical studies, while SI > 50–100 indicates the presence of a wide therapeutic window [7,43].

The SI values obtained for CRG/ACV complex in its liposomal form (SI 128) exceed the threshold of 100. This indicates their high selectivity of action, which is expressed in the effective suppression of HSV-1 replication with minimal cytotoxic effect on cells. The liposomal formulations not only exhibited good antiviral effects when treating infected cells (SI 322) but also demonstrated a higher prophylactic effect than the complex alone. The significantly higher SI values of the liposomal forms indicate that encapsulation of the complex in liposomes not only preserves its antiviral activity but also reduces the cytotoxic effect on host cells. This may be due to changes in the kinetics of interaction with the cell membrane of liposomes and is consistent with data from other studies demonstrating the advantages of liposomal forms [26,44]. The system we developed is fundamentally different from other liposomal acyclovir delivery platforms. Most of the systems presented in the literature (ethosomes, elastic liposomes, multivesicular liposomes, solid lipid nanoparticles) are aimed primarily at improving pharmacokinetic parameters—increasing permeability, prolonging release, increasing the bioavailability of the drug [45,46], whereas our system provides a significant increase in the selective index.

The CRG/ACV complex and its liposomal form may represent new antiviral compounds with a mechanism of action different from those of ACV and its analogues. They may selectively inhibit viral adsorption and replication without harming the host organism, while combining antiviral, anti-inflammatory, and immunomodulatory properties associated with CRG. The obtained results indicate a high potential of the complex, especially its liposomal form, for further preclinical and clinical studies.

## 4. Materials and Methods

### 4.1. Isolation and Analysis of CRGs

The red algae *C. armatus* (Gigartinaceae) samples were collected in the Japan Sea, near the transition between boreal and tropical zones. The presence of male gametophytes was verified based on morphological characteristics and identified via light microscopy. The polysaccharide was extracted using the method described previously [27]. Briefly, after washing the seaweeds with tap water to eliminate excess salt, they were treated with acetone to extract pigments. Dried and ground algae (50 g) were suspended in hot water (1.5 L) for polysaccharide extraction at 90 °C for 2 h, repeated three times. The mixtures were centrifuged, and the supernatants were filtered using a Vivaflow 200 membrane (Sartorius AG, Göttingen, Germany) to eliminate low-molecular-weight compounds. Polysaccharides were precipitated with triple the volume of 96% ethanol to obtain the total polysaccharides (Σ-CRG).

Further, the Σ-CRG were separated into gelling KCl-insoluble and non-gelling KCl-soluble fractions using a 4% *w*/*v* KCl solution. The structure of the fractions was analyzed. To assess the 3,6-anhydrogalactose content, total hydrolysis of CRGs was conducted in 2 M trifluoroacetic acid at 100 °C for 4 h, followed by the formation of aldononitrile acetates [47]. The sulfate content was quantified using turbidimetry [48]. The molecular weight of the polysaccharides was analyzed by HPLC on an Agilent 1100 system (Agilent, Waldbronn, Germany) using a Shodex GS-620 (gel filtration column (Showa Denko, Minato-ku, Japan) and a refractometer (RID G1362A, Agilent, Waldbrom, Germany). A volume of 0.1 M LiNO3 was used as the mobile phase at a flow rate of 0.5 mL/min, with a 50 μL sample injection. Calibration was performed with sulfated dextrans of varying molecular weights (Sigma-Aldrich, St. Louis and Burlington, MA, USA).

### 4.2. FTIR and NMR Spectra

The FTIR spectra for ACV in the KBr pellets, CRG, and CRG/ACV in the films were recorded using Invenio S and Equinox 55 Fourier transform spectrophotometers (Bruker, Hamburg, Germany). OPUS/IR version 7.2 software was used to measure the absorption band frequencies in the IR spectra, with a precision of ≤0.5 cm^−1^. A sample was dissolved in water and dried until a film formed. This film was then placed between two NaCl windows for IR analysis, with normalization performed at ~1070 cm^−1^ (A_1070_ = 1.0).

The ^1^H (500 MHz) and ^13^C (125.75 MHz) NMR spectra were recorded on a DRX-500 spectrometer at 50 °C (Bruker, Hamburg, Germany). The polysaccharides were twice deuterium-exchanged with D_2_O using freeze-drying, then examined in 99.95% D_2_O. Chemical shifts were referenced to acetone (δC 31.45, δH 2.25). Data acquisition and processing were conducted with XWIN-NMR 1.2 software (Bruker, Hamburg, Germany).

### 4.3. Quantum-Chemical Calculations

Quantum-chemical calculations were conducted using density functional theory (DFT) with the B3LYP functional [49] and the 6–31G(d,p) atomic orbital basis set. Gaussian 16W software (Version 1.1, Gaussian Inc., Wallingford, CT, USA, 2019) was employed with default algorithms. Gibbs energy (G) and enthalpy (H) were calculated using the polarized continuum model (PCM) [50]. The main conformer was defined as the one with the highest absolute value of total Gibbs energy (ΔG_main_ = 0.00 kcal/mol). The enthalpy of the carrageenan/acyclovir complexes was estimated by the equation below as the difference between the enthalpy of the H-complex and the sum of the enthalpies of the reactants:

Δ*H* = *H*(complex) − Σ*H*(reactants)

### 4.4. ACV/CRG Complexes

The commercial sodium Acyclovir (cat no. 101931, lot no. 2024H, Sigma-Aldrich, USA) was sourced from Sigma. The CRG was dissolved in deionized water and subsequently filtered through a 0.45 µm membrane. The ACV solution (1 mg/mL) was added to the κ-CRG solution (5 mg/mL). The ACV/CRG complexes were formed by mixing the solutions in specified ratios (1:1 to 1:100 *w*/*w*) and stirring for 24 h at 25 °C.

### 4.5. Liposome Preparation

The liposomes were prepared according to the method described earlier [24]. Briefly, solution of L-α-phosphatidyl choline (Egg chicken, 840051, Avanti Polar Lipids (Alabaster, AL, USA), 100 mg/mL; 0.24 mL) and cholesterol (Sigma Grade ≥ 99%, c 8667, 70 mg/mL; 0.15 mL) in a chloroform–methanol mix (9:1, *v*/*v*) was combined, then evaporated to form a thin film. The film was further dried under vacuum (0.2 Pa) for 2 h, yielding 4 lipid films.

To the dried films, 1 mL of either water (for conventional liposomes), 0.25% Σ-CRG solution (for CRG-containing liposomes), 0.25% ACV solution (for ACV-containing liposomes), or a 1/1 (*w*/*w*) CRG/ACV complex solution (for CRG/ACV-containing liposomes) was added. The test tubes were sonicated twice for 15 min using an ultrasonic bath, then the resulting suspensions were centrifuged for 20 min at 15,000× *g*. The supernatants were removed, and the pellets were re-suspended in 5 mL of water, followed by three wash cycles.

After the final centrifugation, the pellets were re-suspended in 2 mL of water and sonicated for 30 min to reduce liposome size, then lyophilized in vials. For analysis, the lyophilized liposomes were hydrated by sonication in water for 30 min at a concentration of 1 mg/mL. To assess the ACV content, several milligrams of lyophilized liposomes were mixed with 1 mL of water and 1 mL of n-butanol, sonicated for 30 min, and centrifuged for 20 min at 15,000× *g*, using the lower aqueous layer for analysis. The ACV concentration was determined by UV spectroscopy at 254 nm, and CRG was analyzed using the Taylor–Blue method at 535 nm [51].

### 4.6. Dynamic Light Scattering (DLS) and Electrophoretic Analysis

The sizes and ζ-potentials of the initial polysaccharides and CRG/ACV complex in aqueous solution, as well as liposomes containing the CRG/ACV complex, were measured using a ZetaSizer NanoZS system (Malvern, Worcestershire, UK) at 633 nm. Samples were left for 1 h to let large aggregates settle, reducing interference, even when their concentration was minimal. Measurements were conducted at 25 °C. Hydrodynamic diameters were automatically calculated from the autocorrelation function using the instrument’s software, while ζ-potentials were derived from electrophoretic mobility values via the Henry equation [52]. All measurements were conducted in triplicate.

### 4.7. Complex Formation with Mucin

To prepare the complex, 1 mL of a mucin solution (1 or 5 mg/mL) was combined with 1 mL of the CRG/ACV complex or liposomes containing the complex (C = 0.5 mg/mL) in water. The mixture was incubated (1 or 18 h) prior to analysis.

### 4.8. Determination of Mucoadhesive Properties of CRG/ACV Complex by the Mechanical Method

The mucous tissue from the fresh-frozen inner surface of the mucous membrane of the nasal cavity of a pig was used as a model and the mechanical properties were determined at five points for each sample as described by us earlier [25]. The work of mucoadhesion (W_adg_) is a function of stress and strain, calculated by the area under the curve σ = f (d). The force of adhesion was calculated using Exponent Software (Version V6.1.5.0). Each series consisted of 7 measurements. The result is presented as a mean with a 95% confidence interval error (CI_95_ = 1.95 * SE).

### 4.9. Antiherpetic Activity

#### 4.9.1. Virus and Cell Culture

The HSV-1 strain L2 was sourced from the G.P. Somov Institute of Epidemiology and Microbiology, Rospotrebnadzor. The virus was cultivated in Vero cells (kidney epithelial cells from African green monkey *Chlorocebus* sp.) using Dulbecco’s Modified Eagle’s Medium (DMEM, Biolot, St. Petersburg, Russia) supplemented with 10% fetal bovine serum (FBS, Biolot, St. Petersburg, Russia) and 100 U/mL of gentamicin (Dalkhimpharm, Khabarovsk, Russia), incubated at 37 °C in CO_2_. For maintenance, the FBS concentration was reduced to 1%. The cell density for all experiments was maintained at 10^4^ cells/mL.

#### 4.9.2. Cytotoxicity and Anti-HSV-1 Activity of CRG/ACV Complex and Its Liposomal Form

Cytotoxicity of the CRG/ACV complex was assessed using the MTT assay on Vero cells, following established protocols [37,53]. Briefly, Vero cell monolayers (1 × 10^4^ cells/well) in 96-well plates were treated with varying concentrations of compounds (1–2000 µg/mL) for 72 h at 37 °C in 5% CO_2_, with untreated cells as controls. An MTT (methylthiazolyltetrazolium bromide, Sigma, St. Louis, MO, USA) solution (5 mg/mL) was added, and after 2 h of incubation, the absorbance was read at 540 nm (Labsystems Multiskan RC, Vantaa, Finland). Cytotoxicity was quantified as the CC_50_, the concentration reducing cell viability by 50%, with experiments conducted in triplicate across three separate tests. To evaluate the inhibitory effects against HSV-1 in Vero cells, a cytopathic effect (CPE) inhibition assay was performed. Compounds were tested at concentrations from 1 to 500 µg/mL against an infectious dose of HSV-1 (100 TCID_50_/mL). Two application methods were explored, with each trial repeated independently three times.

Pre-treatment of cells: Cells were treated with compounds for 2 h at 37 °C, washed, and then infected with the virus for 1 h. After washing off the unabsorbed virus, the cells were maintained until CPE developed.

Post-infection treatment: Cells were first infected with the virus (100 TCID_50_/mL) for 1 h, washed, and then treated with compounds before monitoring for CPE.

The efficacy of the compounds in inhibiting HSV-1 was determined using the protection index (PI), calculated by Pauwels’ formula [54] as

$$PI(C)=\frac{D_{ci}-D_{i}}{D_{0}-D_{i}}\cdot 100\%,$$

where D_0_ is the optical density for uninfected cells without treatment, D_i_ is for infected cells without treatment, and D_ci_ is for infected cells treated with the compound. The 50% inhibitory concentration (IC_50_) was derived using linear–logarithmic interpolation.

$$PI\left({IC}_{50}\right)=50\%$$

Effectiveness was further characterized using the selectivity index (SI):

$$SI=\frac{{CC}_{50}}{{IC}_{50}}$$

### 4.10. Statistical Analysis

Statistical analysis was conducted with Statistica 10.0 software (StatSoft, Inc., Tulsa, OK, USA). All measurements were taken in triplicates. CC_50_ and IC_50_ values were derived from linear–logarithmic interpolation of the dose–response curves. Results were reported as mean ± standard deviation (SD) and were compared using ANOVA, with significance set at *p* < 0.05. Data analysis was executed using Statistica 6.0 software (StatSoft, USA).

## Figures and Tables

**Table 1:** ζ-potential and Z-average measurements of CRG solutions, their complexes with ACV and CRG/ACV-loaded liposomes.

	Z-Average, d, nm		Charge, mV	
Samples	Without Mucin	with Mucin	Without Mucin	with Mucin
ACV Sigma	-		−6.9 ± 2.9%	
Mucin	1909.8 ± 4.0		−26.1 ± 0.8	
κ-CRG	614.6 ± 88.5		−85.3 ± 0.8	
κ-CRG/ACV 10: 1	1076 ± 83		−74.1 ± 0.8	
Σ-CRG	683.5 ± 24.3	774.8 ± 14.3	−68.7 ± 0.7	−36.6 ± 0.8
Σ CRG/ACV 10:1	1097.0 ± 39.15	1001.3 ± 188.6	−63.3 ± 1.0	−41.6 ± 0.7
Control liposomes	363.8 ± 8.7 (62%) 107.6 ± 12.0 (38%)	605.6 ± 140.7 (68%) 115.1 ± 16.2 (32%)	−14.5 ± 1.2	−21.5 ± 0.5
Liposomes with Σ-CRG/ACV 10:1	436.3 ± 63.5 (66%) 124.0 ± 17.0 (34%)	421.2 ± 68.9 (88%) 76.7 ± 13.6 (12%)	−28.4 ± 1.4	−31.0 ± 0.5
Lyophilized liposomes with Σ-CRG/ACV 10:1	598.2 ± 80.6 (77%) 107.0 ± 11.6 (23%)	-	−25.6 ± 1.1	

**Table 2:** Anti-HSV-1 activity of the complex Σ-CRG/ACV and its liposomal form.

Compounds	CC_50_ (µg/mL)	Pre-Treatment of Cells		Treatment of Infected Cells	
		IC_50_ (µg/mL)	SI	IC_50_ (µg/mL)	SI
Σ-CRG	>2000	18 ± 3	111 ± 14	77 ± 11	26 ± 3.4
ACV	>1000	no activity		1.9 ± 0.3	609 ± 67
Σ-CRG/ACV 10:1	>1000	35 ± 4	28 ± 3	1.8 ± 0.2	555 ± 61
Σ-CRG/ACV 100:1	>1000	26 ± 3	38 ± 5	16 ± 2	63 ± 8
Liposomes with Σ-CRG	>1000	5.3 ± 0.7	189 ± 21	not studied	
Liposomes with ACV	>1000	no activity		2.9 ± 0.4	344 ± 38
Liposomes with Σ-CRG/ACV	>1000	7.8 ± 0.9	128 ± 14	3.1 ± 0.4	322 ± 35
Empty liposomes	>2000	no activity		no activity	

Note: CC_50_—50% cytotoxic concentration of the compound (μg/mL); IC_50_—50% virus-inhibitory concentration of the compound (μg/mL); SI—selective index of the compound (SI = CC_50_/IC_50_). Liposomes with Σ-CRG (contents of the Σ-CRG—0.28%); liposomes with ACV (contents of the ACV—0.28%); liposomes with Σ-CRG/ACV (1:1) (contents of the Σ-CRG and ACV: 0.24% and 0.24%). The antiviral activity of liposomes was assessed based on their active substance content. Data are expressed as means ± SD of three independent experiments.

## Data Availability

The original contributions presented in this study are included in the article/Supplementary Material. Further inquiries can be directed to the corresponding author.

## Supplementary Materials

The following supporting information can be downloaded at https://www.mdpi.com/article/10.3390/ijms27083367/s1.
