# Supplementary material for: Carrageenan–Acyclovir Complex and Its Liposomal Form
Source: Int J Mol Sci. 2026 Apr 9;27(8):3367. doi: 10.3390/ijms27083367 (PMC13115838; doi:10.3390/ijms27083367)
Supplement: Supplementary file 1 [file ijms-27-03367-s001.zip › ijms-4200830-supplementary.pdf]

Supplementary

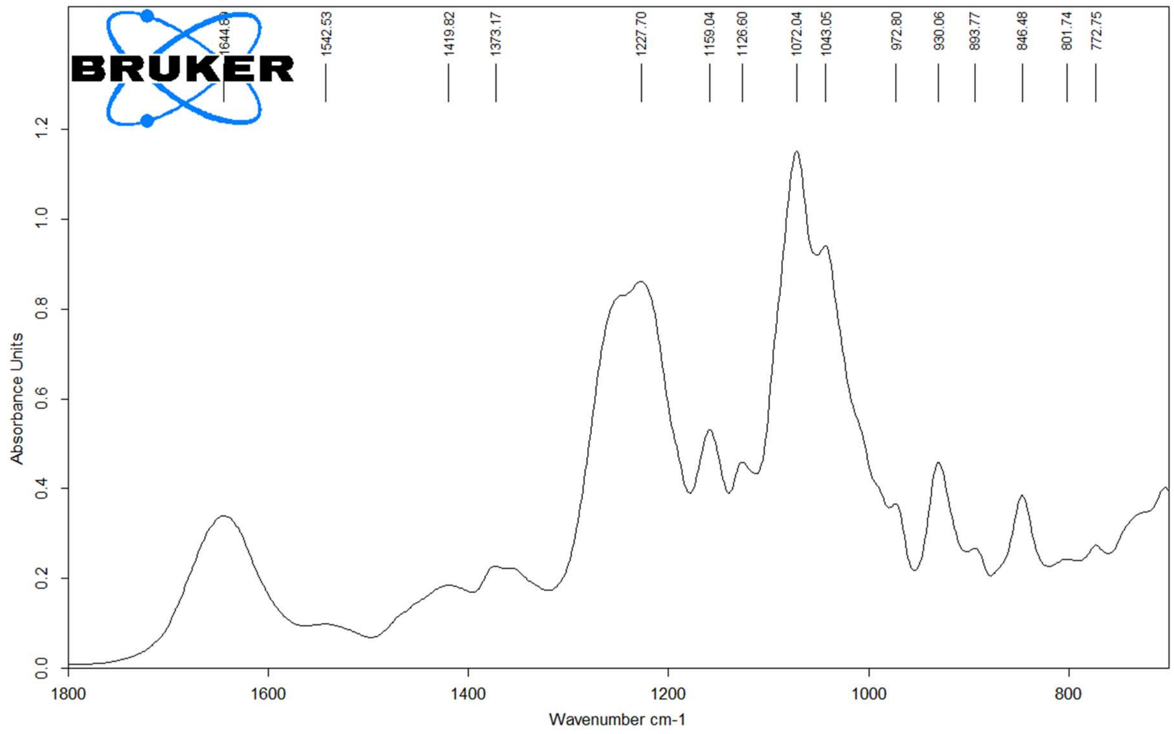

Figure S1. IR-spectrum of κ-CRG (KCl-gelling polysaccharide)

Table S1. ^{13}C and ^{1}H NMR data of gelling polysaccharide

Type of CRG	Unit	^{13}C/^{1}H chemical shift (ppm)					
		C-1/H-1	C-2/H-2	C-3/H-3	C-4/H-4	C-5/H-5	C-6/H-6
**κ-CRG**	G4S	103.1/4.60	70.4/3.60	79.1/3.99	74.7/4.87	75.5/3.80	62.0/3.80
	DA	95.9/5.10	70.5/4.14	79.8/4.52	79.1/4.62	77.1/4.66	69.5/4.20

Table S2. Characteristic of CRGs from *Chondrus armatus* [27]

	Structure of disaccharide units		Content , dry weight %			MW, kDa
Samples	3-linked	4-linked	Gal	3,6-AnGal	SO_4^{2-}	
κ-CRG	G4S	DA	37.1	33.0	22.0	560
Σ -CRG	G4S<br>G2S	DA<br>D2S,6S	40.1	16.2	27.1	185

Note: G2S: 1,3-β-D-galactose 2-sulfate; DA: 1,4-3,6-anhydro-α-D-galactose; G4S: 1,3-β-D-galactose 4-sulfate; D2S,6S: 1,4-α-D-galactose 2,6-disulfate. Gal: galactose; 3,6-AnGal: 3,6-anhydrogalactose; SO_4^{2-} : sulfate group

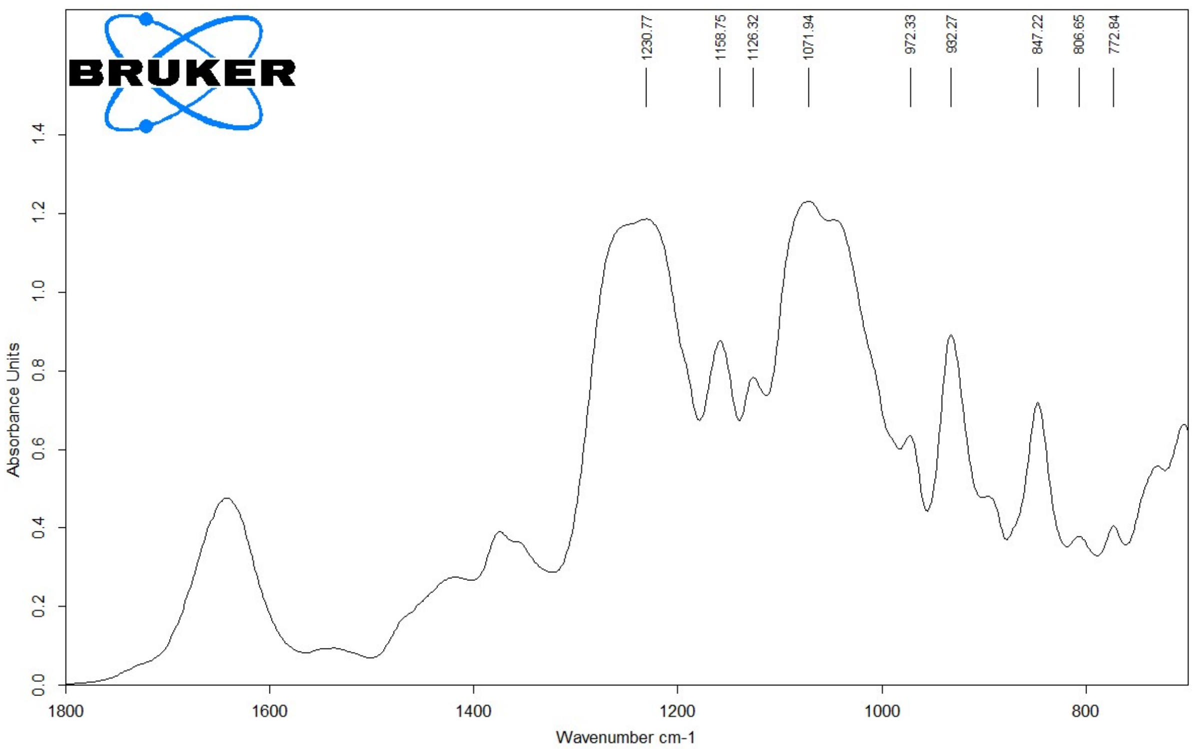

Figure S2. IR-spectrum of Σ-CRG

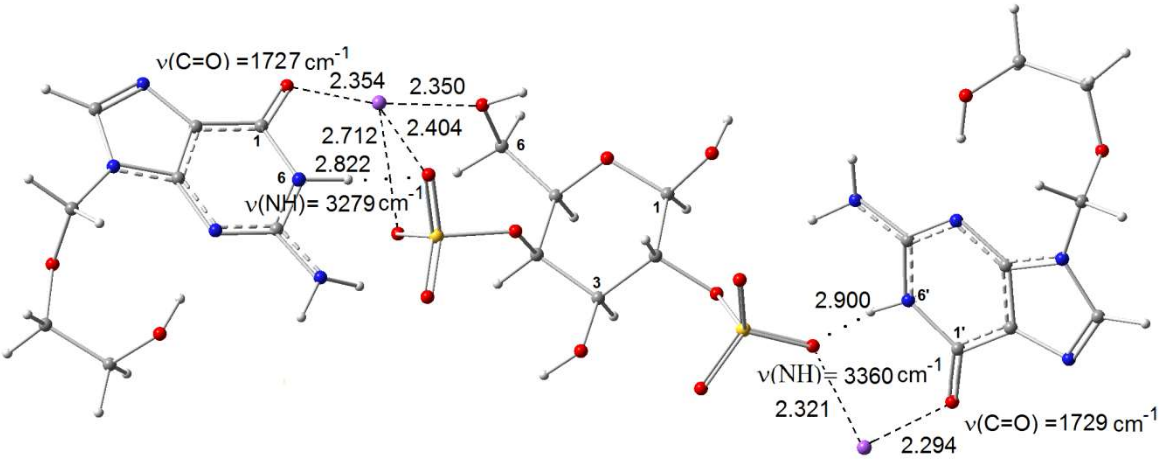

Figure S3. Theoretical model of the “ λ –CRG/ ACV complex $-\Delta H = 19.6$ kcal/mol

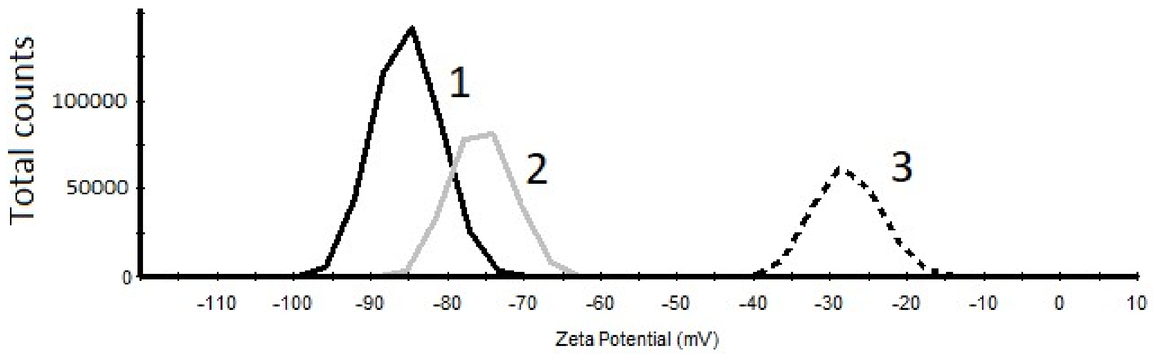

Figure S4. Zeta potential distribution: 1 – κ-CRG; 2 and 3 – mixtures κ-CRG with ACV at ration 10:1(2) and 100:1(3) (w/w)
